# Hidden Microatelectases Increase Vulnerability to Ventilation-Induced Lung Injury

**DOI:** 10.3389/fphys.2020.530485

**Published:** 2020-09-18

**Authors:** Karolin Albert, Jeanne-Marie Krischer, Alexander Pfaffenroth, Sabrina Wilde, Elena Lopez-Rodriguez, Armin Braun, Bradford J. Smith, Lars Knudsen

**Affiliations:** ^1^Institute of Functional and Applied Anatomy, Hannover Medical School, Hanover, Germany; ^2^Fraunhofer Institute for Toxicology and Experimental Medicine, Hanover, Germany; ^3^Biomedical Research in Endstage and Obstructive Lung Disease Hannover (BREATH), German Center for Lung Research, Hanover, Germany; ^4^Institute for Functional Anatomy, Charité, Berlin, Germany; ^5^Department of Bioengineering, College of Engineering, Design and Computing, University of Colorado Denver, Anschutz Medical Campus, Aurora, CO, United States

**Keywords:** microatelectases, alveolar interdependence, ventilation-induced lung injury, surfactant, blood-gas barrier

## Abstract

Mechanical ventilation of lungs suffering from microatelectases may trigger the development of acute lung injury (ALI). Direct lung injury by bleomycin results in surfactant dysfunction and microatelectases at day 1 while tissue elastance and oxygenation remain normal. Computational simulations of alveolar micromechanics 1-day post-bleomycin predict persisting microatelectases throughout the respiratory cycle and increased alveolar strain during low positive end-expiratory pressure (PEEP) ventilation. As such, we hypothesize that mechanical ventilation in presence of microatelectases, which occur at low but not at higher PEEP, aggravates and unmasks ALI in the bleomycin injury model. Rats were randomized and challenged with bleomycin (B) or not (H = healthy). One day after bleomycin instillation the animals were ventilated for 3 h with PEEP 1 (PEEP1) or 5 cmH_2_O (PEEP5) and a tidal volume of 10 ml/kg bodyweight. Tissue elastance was repetitively measured after a recruitment maneuver to investigate the degree of distal airspace instability. The right lung was subjected to bronchoalveolar lavage (BAL), the left lung was fixed for design-based stereology at light- and electron microscopic level. Prior to mechanical ventilation, lung tissue elastance did not differ. During mechanical ventilation tissue elastance increased in bleomycin-injured lungs ventilated with PEEP = 1 cmH_2_O but remained stable in all other groups. Measurements at the conclusion of ventilation showed the largest time-dependent increase in tissue elastance after recruitment in B/PEEP1, indicating increased instability of distal airspaces. These lung mechanical findings correlated with BAL measurements including elevated BAL neutrophilic granulocytes as well as BAL protein and albumin in B/PEEP1. Moreover, the increased septal wall thickness and volume of peri-bronchiolar-vascular connective tissue in B/PEEP1 suggested aggravation of interstitial edema by ventilation in presence of microatelectases. At the electron microscopic level, the largest surface area of injured alveolar epithelial was observed in bleomycin-challenged lungs after PEEP = 1 cmH_2_O ventilation. After bleomycin treatment cellular markers of endoplasmic reticulum stress (p-Perk and p-EIF-2α) were positive within the septal wall and ventilation with PEEP = 1 cmH_2_O ventilation increased the surface area stained positively for p-EIF-2α. In conclusion, hidden microatelectases are linked with an increased pulmonary vulnerability for mechanical ventilation characterized by an aggravation of epithelial injury.

## Introduction

The clinical picture encompassing refractory blood deoxygenation, decrease in respiratory system’s compliance and bilateral infiltrates in conventional chest X-ray has been described originally as acute respiratory distress syndrome (ARDS) by Ashbaugh et al. (1967). For those patients suffering from ARDS, mechanical ventilation represents the decisive lifesaving therapeutic intervention. On the other hand, mechanical ventilation has also been identified to be an independent trigger for the progression of acute lung injury (ALI) through a process referred to as ventilator-induced lung injury (VILI) (Slutsky and Ranieri, 2013) and a substantial fraction of ARDS patients develop the complete picture of the ARDS during mechanical ventilation (Nieman et al., 2015). Moreover, mechanical ventilation in presence of surfactant abnormalities plays a central role in the development of an ARDS/ALI (Albert, 2012; Rühl et al., 2019).

The underlying pathomechanisms involve abnormalities in alveolar micromechanics. Alveolar micromechanics can be defined as the dynamic changes of alveoli in size and shape during a respiratory cycle (Knudsen and Ochs, 2018). In healthy lungs the alveolar airspaces are stabilized by connective tissue elements located within the inter-alveolar septal walls as well as the intra-alveolar surfactant layer at the gas-liquid interface which reduces surface tension during expiration (Wilson and Bachofen, 1982; Knudsen and Ochs, 2018). In presence of a functional surfactant system the stresses are balanced and roughly homogenously distributed within the lung (Mead, 1961; Mead et al., 1970). During a respiratory cycle the strains and stresses acting on the inter-alveolar septa (e.g., the blood-gas barrier) are therefore minimized (Knudsen and Ochs, 2018). Direct or indirect lung injury results in inflammation, edema formation, and surfactant dysfunction that cause severe organ dysfunction. These pathologies introduce heterogeneous ventilation and abnormal alveolar micromechanics. Surfactant dysfunction results in intra-tidal alveolar recruitment and derecruitment (Schiller et al., 2001), an abnormality which, in presence of high surface tension, injures the epithelium and is therefore named atelectrauma (Slutsky and Ranieri, 2013; Cressoni et al., 2017; Rühl et al., 2019). The inter-alveolar walls of those alveoli which remain open are subject to increased strain and overdistension. This volutrauma further injures the very thin and delicate blood-gas barrier by rupturing the endothelial or alveolar epithelial cells (Dreyfuss and Saumon, 1998; Vlahakis et al., 2002; Cong et al., 2017). A more insidious mechanism of volutrauma results from the concept of alveolar interdependence (Mead et al., 1970; Makiyama et al., 2014). In this situation, microatelectases act as stress concentrators that impose tethering forces on surrounding inter-alveolar septal walls so that these areas of lung parenchyma are prone to overdistension and therefore volutrauma (Albert, 2012; Albert et al., 2019).

Ruptures of the plasma membrane of alveolar epithelial cells increase cytoplasmic Ca^2+^ levels which are responsible for activation of the cellular integrated stress response (ISR) (Dolinay et al., 2017, 2018). Stretch-induced ISR is initiated by Ca^2+^ dependent auto-phosphorylation of protein kinase RNA-like endoplasmic reticulum kinase (Perk). Phosphorylated Perk (p-Perk) moreover phosphorylates eukaryotic translation initiating factor 2α (EIF-2α), a step critical to switch off the protein biosynthesis machinery of the cell (Harding et al., 2000). If the ISR lingers on then cell death is initiated by selective expression of CCAAT/Enhancer binding Protein homologous Protein (CHOP) (Günther et al., 2012; Dolinay et al., 2018).

Intratracheal instillation of bleomycin is a common animal model of dose-dependent direct acute lung injury and fibrosis (Bonniaud et al., 2018). We have previously characterized the alveolar micromechanics of this model in detail during disease progression. The first abnormality observed after 24 h was a slight surfactant dysfunction linked with pathological alterations in alveolar micromechanics (Lutz et al., 2015; Steffen et al., 2017; Knudsen et al., 2018) where a decrease in airway opening pressure below 5 cmH_2_O on expiration resulted in a derecruitment of alveoli. At a light microscopic level, further pathologies including edema formation or inflammation were virtually absent (Lutz et al., 2015; Knudsen et al., 2018). Moreover, at this very early stage of injury the lung mechanical properties, including organ scale tissue elastance within a range of PEEP levels from 1 to 5 cmH_2_O as well as oxygen saturation, did not significantly differ from healthy subjects. Due to the fact that pressures needed to recruit alveoli were much higher than those needed to prevent their decruitment, computational simulations of alveolar micromechanics suggested that in this model there was only little intra-tidal alveolar recruitment and derecruitment during ventilation with PEEPs between 0 and 15 cmH_2_O and a tidal volume of 10 ml/kg bodyweight (Knudsen et al., 2018). Instead, ventilation with PEEP below 5 cmH_2_O was associated with permanent alveolar derecruitment (=microatelectases) combined with increased tidal alveolar volume changes indicating an increased dynamic alveolar strain. In addition, the open alveoli were characterized by lower individual elastance, a phenomenon explainable by outward tethering forces imposed by nearby derecruited alveoli. Based on these observations, we considered the bleomycin-injured lung to be at risk for VILI when PEEP is below 5 cmH_2_O due to the presence of microatelectases. Since neither tissue elastance nor blood oxygenation differed from healthy controls there were no clinical signs of ALI at the organ scale. The microatelectases were therefore considered to be hidden.

In order to investigate the hypothesis whether these hidden microatelectases are linked with an increased susceptibility to VILI, bleomycin challenged lungs were ventilated with a PEEP of 1 (=microatelectases present) or 5 cmH_2_O (=microatelectases absent) and compared to healthy controls. After a period of 3 h of mechanical ventilation ALI was unmasked in bleomycin challenged lungs if ventilated with PEEP of 1 but not 5 cmH_2_O based on tissue elastance measurements, alveolar edema, ultrastructural signs of injury of alveolar epithelial cells and markers of ISR.

## Materials and Methods

### Subjects and Study Design

The animal experiments were approved by the Niedersächsisches Landesamt für Verbraucherschutz und Lebensmittelsicherheit (LAVES, Oldenburg, Lower Saxony, Germany, approval number 17/2068) which house the German equivalent of an institutional animal care and use committee, according to the European Animal Welfare Regulations. In total 50 male Fisher 344/DuCrl rats (Charles River, Sulzfeld, Germany) aged 11 to 13 weeks with body weights ranging between 208 and 250 g were randomly assigned to a healthy group (H; *n* = 23) and a bleomycin-injured group (B; *n* = 27). The bleomycin group received intratracheal instillation of 4.5 U/kg bodyweight of bleomycin dissolved in 0.9% NaCl solution. One day later animals were further randomized to the subgroups which were either not ventilated (groups H/No ventil, *n* = 9, and B/No ventil, *n* = 8) or ventilated with PEEP = 1 cmH_2_O (groups H/PEEP1, *n* = 7, and B/PEEP1, *n* = 9) or PEEP = 5 cmH_2_O (groups H/PEEP5, *n* = 7, and B/PEEP5, *n* = 10). The animals were anesthetized by intraperitoneal injection of Ketamine (80 mg/kg bodyweight) combined with Xylazine (5 mg/kg bodyweight) and tracheotomized. Those animals assigned to “no ventilation” were directly subjected to bronchoalveolar lavage, and instillation fixation or vascular perfusion fixation as outlined below. Those animals assigned to ventilation groups were ventilated with room air, initially with PEEP = 3 cmH_2_O, tidal volume = 10 ml/kg bodyweight and an inspiratory-to-expiratory ratio of 1:2. The respiratory rate was 90/min. The choice of these parameters was geared to previous studies applying similar volumes per minute and kg bodyweight during mechanical ventilation (Allen et al., 2005). The baseline lung mechanical properties were measured using derecruitability tests administered with a *newFlexiVent-System* (SCIREQ^®^, Montreal). These derecruitability tests included 2 deep inflations consisting of a continuous ramp of airway opening pressure from 3 to 30 cmH_2_O over 3 s and an end-inspiratory plateau at 30 cmH_2_O for 3 s. The deep inflations, or recruitment maneuvers, were followed by 10 repetitive forced oscillation perturbations (FOT) over a period of 4.5 minutes with the first FOT performed 2 seconds after the recruitment. The impedance spectra measured by FOT over a frequency range between 1 and 20.5 Hz were fit to the constant-phase model in order to calculate tissue elastance H (Hantos et al., 1992). The first tissue elastance value of this derecruitability test was referred to as H1 and reflected lung mechanical properties at the highest degree of recruitment. The mean of the last three measurements of the tissue elastance was referred to as Hs and reflected steady state conditions between 3.5 and 4.5 min after recruitment. The increase of tissue elastance over time after recruitment maneuver has been linked with progressive derecruitment of distal airspaces and was calculated as ΔH = Hs − H1. After assessments of baseline lung mechanical properties, the PEEP was set to either 1 cmH_2_O (PEEP1 groups) or 5 cmH_2_O (PEEP5 groups). Anesthesia was maintained by inhalation narcosis using 1% isoflurane in room air combined with subcutaneous injection of 2 mg/kg bodyweight butorphanol (Torbugesic^®^). The tidal volume, inspiratory-to-expiratory ratio and respiratory rate remained unchanged. Derecruitability tests were performed every 60 min as described above with a PEEP, and FOT onset pressure, set according to the ventilation pattern (1 or 5 cmH_2_O). After 3 h of mechanical ventilation a final derecruitability test was conducted at PEEP = 3 cmH_2_O. Since the PEEP value has an impact on the lung mechanical properties (Knudsen et al., 2018) the measurements before and after the 3 h period of either PEEP = 1 or 5 cmH_2_O ventilation could be compared between study groups so that differences in tissue elastance reflected the degree of lung injury. Monitoring of animals during mechanical ventilation was based on transcutaneous pulse oximetry using a rodent oximeter (MouseOx Plus, Rat Foot Sensor, Starr Life Sciences Corp., United States) and the oxygen saturation as well as heart rate was documented every 30 min. In addition the pressure in the ventilator cylinder (Pcycl) of the flexiVent system and airway opening at the expiratory limb (Pao) was recorded continuously in *n* = 3 animals per experimental group to describe the time course of the peak inspiratory pressure (PIP) during mechanical ventilation.

### BAL and Tissue Processing

After ventilation, all rats were euthanized with Ketamine (80 mg/kg bodyweight) combined with Xylazine (5 mg/kg bodyweight, Rompun^®^) and the opening of the abdominal aorta. Except for 1–2 lungs per study group all were subjected to BAL and instillation fixation. For this purpose, the right lungs received three repetitions of a 4 ml bronchoalveolar lavage (BAL) using 0.9% sodium chloride (NaCl). Left lungs were fixed by instillation fixation (1.5% Paraformaldehyde, 1.5% Glutaraldehyde, 0.15 M HEPES buffer) at a hydrostatic instillation pressure of 25 cmH_2_O. The lung volumes of fixed lungs were determined before a systematic uniform sampling for light and electron microscopic assessments was performed based on established methods (Tschanz et al., 2014). This sampling process resulted in 5–6 slices sampled for analyses at light microscopical level and at least 6 tissue blocks sampled for analyses at electron microscopic level. Samples were embedded in Technovit 8100 (Kulzer GmbH, Wehrheim, Germany) or epoxy resin (Epon^®^, Polyscience, Hirschberg an der Bergstraße, Germany) for light and electron microscopy, respectively (Mühlfeld et al., 2013). Within each study group the remaining lungs (*n* = 1–2) were fixed by vascular perfusion fixation at an end-inspiratory hold during PEEP = 1 cmH_2_O ventilation using 0.1% glutaraldehyde and 4% paraformaldehyde in 0.2 M Hepes buffer solution. This “fixation from behind” allows assessment of the air-filled lung and therefore the effects of high surface tension on lung structure, e.g., the occurrence of microatelectases or intra-alveolar edema formation, are maintained. This fixative has also been used for immunolabeling since it maintains antigenicity and molecular identity of cells (Hsia et al., 2010). Perfusion fixed lungs were sampled for Technovit embedding and structural investigations as well as O.C.T. (optical cutting temperature) embedding for cryosections and immunolabeling of cellular stress markers.

### BAL Analyses

For protein and cellular analysis, the BAL components were separated by centrifugation. The protein concentration inside the BAL fluid was analyzed by a protein assay (Pierce^TM^ BCA Protein Assay, Fisher Scientific, Hampton, VA, United States) based on the manufacturer’s instructions. Albumin concentration in the BAL was measured via ELISA (Rat Albumin ELISA Kit, Bethyl Laboratories, Montgomery). The BAL samples were diluted 1:1000 and compared to the standard curve according to the manufacturer’s protocol. For cellular analysis, the BAL cells were separated from the rest of the BAL via centrifugation. Cytospin slides (Thermo Shandon Cytospin 3, Marshall Scientific, Hampton VA, United States) were prepared with 50.000 cells per BAL diluted in 100 μl phosphate buffer (PBS). Lymphocytes, macrophages and neutrophilic granulocytes were differentiated after staining the Cytospins with DiffQuik. Using Visiopharm^®^ Software a systematic uniform area sampling was performed and at least 100 to 200 cells were categorized and counted using a primary magnification of 40×. Furthermore, we assessed the concentration of IL-6 within the BAL using an ELISA Kit (DuoSet^®^ ELISA Rat IL-6, R&D Systems, Minneapolis, MI, United States). We utilized 96 well-plates and photometric measurements for cytokine concentration. Calculations were made with Magellan Software.

### Design-Based Stereology

For stereology, lungs fixed by airway instillation were used. Initially we performed a volumetry based on the Archimedes principle for the whole left lung to obtain the volume of the reference space, the lung volume, for further structural assessments (Scherle, 1970). Embedded tissue for light microscopic analyses was cut into 1.5 μm sections by a microtome (Leica RM 2165, Leica Biosystems, Wetzlar, Germany). Afterward, a toluidine blue staining was performed. The slides for light microscopy were scanned (AxioScanZ.1) and 10% of the area of each slide was randomly sampled with the use of Visiopharm^®^ Software to obtain between 60 and 200 representative fields of view per lung. The pictures were examined by using the STEPanizer stereology tool (Tschanz et al., 2011) or the newCAST Visiopharm software. Following a cascade sampling design (Ochs, 2006) non-parenchyma (nonpar) was initially differentiated from parenchyma (par) and volume fractions of these compartments (V_V_(par/lung) and V_V_(nonpar/lung)) within the lung were determined by point counting. Parenchyma was defined as regions of the lung that contribute to diffusive gas exchange so that this compartment included alveolar and ductal airspaces as well as interalveolar septal walls. Non-parenchyma included the conducting airways (bronchi and bronchioles), larger vessels (excluding capillaries and corner vessels within the septa) and the peri-bronchiolar-vascular connective tissue. In a second step the components within the non-parenchyma and parenchyma were further differentiated by means of point-counting. Taking the non-parenchyma compartment as reference space, the volume fractions within non-parenchyma of conducting airways (V_V_(airway/nonpar)), larger vessels (V_V_(ves/nonpar)) and peri-bronchiolar-vascular connective tissue (V_V_(pbvtis/nonpar)) were determined. Within parenchyma, alveolar (V_V_(alv/par)) and ductal airspaces (V_V_(duct/par)) as well as interalveolar septa (V_V_(sep/par)) were distinguished. Volume fractions were, in general, calculated by dividing the number of test points falling on the structure of interest (e.g., non-parenchyma) and the test points hitting the reference space (e.g., the total lung volume). Volume fractions were converted to absolute volumes of the structures of interest per lung by multiplication with the volume of the reference space. In addition, the surface area density of alveolar walls within the lung parenchyma (S_V_(alv/par)) was determined by intersection counting based on established methods (Knudsen et al., 2010). The surface density was converted into a total surface area per lung (S(alv,par)) by multiplication with the reference volume, in this case the volume of lung parenchyma (V(par,lung)). For all structures of interest between 100 and 200 counting events per lung were generated since this has been shown to result in a sufficiently high precision of stereological estimators (Gundersen and Jensen, 1987). The mean arithmetic thickness of interalveolar septal walls (t(sep)) was calculated as a volume-to-surface ratio (Mühlfeld and Ochs, 2013).

Electron microscopy (FEI Morgagni transmission electron microscope, Eindhoven, The Netherlands) was used to investigate the health of the alveolar epithelium based on ultrastructural criteria (Dreyfuss and Saumon, 1998; Fehrenbach et al., 1999; Lutz et al., 2015; Rühl et al., 2019). The samples for electron microscopy were embedded in epoxy resin and cut into 80 nm sections (Ultracut S, Leica Reichert, Wetzlar, Germany). Six sections per lung were analyzed and 100 to 200 fields of view per lung were imaged within a systematic uniform area sampling process (Tschanz et al., 2014). Test lines were superimposed on each image and intersections of the test line with the epithelial basal lamina were counted. At each intersection, the covering epithelial cell was categorized as alveolar epithelial type I (AEI) or type II (AE2) cell. Based on ultrastructural criteria, the corresponding epithelial cell was further categorized as being either “intact” or “injured.” The criteria for an injured epithelial cell were as follows: clearing and swelling of the cytoplasmic ground substance (in relation to the other cells in the septum), blebbing and fragmentation of cells, or denudation of the epithelial basal lamina. The surface densities of the basal lamina covered by the differently categorized epithelial cells were then calculated. By multiplication of the surface densities with the reference volume, in this case the total volume of interalveolar septa (V(sep,par)), absolute surface areas of the basal lamina covered by intact (S(AE1_intact,par); S(AE2_intact,par)) or injured AE1 or AE2 cells (S(AE1_injure,par); S(AE2_injure,par) could be determined (Lutz et al., 2015; Rühl et al., 2019). The light and electron microscopic images used for design-based stereology in this study are available on https://zenodo.org (Albert et al., 2020, 10.5281/zenodo.3738928).

### Immunohistochemistry and Immunofluorescence of Cellular Stress Markers

Cryosections with a thickness of 5 μm were cut for immunohistochemistry of cellular stress markers (p-Perk and p-EIF-2α). Sections were pre-treated for 7 min at 37°C with 0.01% trypsin in 0.1% CaCl and repetitively washed with phosphate buffer (PBS). Endogenous peroxidases were blocked with 0.06% hydrogen peroxide in PBS. Primary antibodies (Rabbit anti p-EIF-2α, Cell Signaling Technologies #3398; Rabbit anti p-Perk, Cell Signaling Technologies #3179) were diluted 1:200 in PBS and used for incubation at 4°C in a moist chamber overnight. Staining with an isotype control antibody (Rabbit mAb, IgG, Cell Signaling Technologies, #3900) were used as a negative control according to the manufacturer’s recommendations. Biotinylated mouse-anti-rabbit antibody was used as the secondary antibody. For detection, a streptavidin – HRP –DAB reaction system was applied and the color development observed. Counterstaining was carried out by means of hematoxylin. For quantification of the percentage of the alveolar surface area covered by p-EIF-2α positive cells a systematic uniform area sampling was performed on four sections per group to obtain at least 200 pictures. Test-lines were randomly projected on the fields of view and intersections of the test lines with brown or not brown colored surfaces were counted. The surface fraction of brown colored surface after p-EIF-2α (S_S_(pEIF2α/sep)) staining was calculated as the ratios of intersections with brown surfaces and all surfaces.

### Statistics

Statistical analysis of the data was done using *GraphPad Prism Version 7* (GraphPad Software, San Diego, CA, United States). A two-way ANOVA was used to take the influencing factors “pre-treatment” (Bleo) and “ventilation pattern” (vent: no ventilation vs. PEEP 1 vs. PEEP 5) or “duration of ventilation” as well as their interaction into account. In case of significant differences based on the two-way ANOVA the Bonferroni *post hoc* test for multiple comparisons and adjustment of the p-level was added. Correlation analyses were performed using Pearson’s test. In general, *p* values below 0.05 were considered as statistically significant. The raw data measured during mechanical ventilation while performing the FOT perturbations were fit to the constant phase model. If the coefficient of determination of the model fit to the raw data (impedance spectra) was below 0.9 the data were excluded from further consideration.

## Results

### Effects of Mechanical Ventilation on Lung Function and Mechanics

During mechanical ventilation oxygen saturation (SO_2_) was measured as a functional parameter. No significant differences could be detected between the study groups both at the beginning and at the end of the 3 h period of mechanical ventilation. Initially, the mean and standard deviation of SO_2_ were 98.8% (0.3%), 98.8% (0.6%), 98.8% (1.3%), and 98.6% (1.5%) for H/PEEP1, H/PEEP5, B/PEEP1 and B/PEEP5, respectively. At the end of the mechanical ventilation SO_2_ data were 98.7% (0.72%), 97.3% (1.6%), 96.2% (2.5%), and 97.7% (1.3%) for H/PEEP1, H/PEEP5, B/PEEP1, and B/PEEP5, respectively.

**FIGURE 1:**
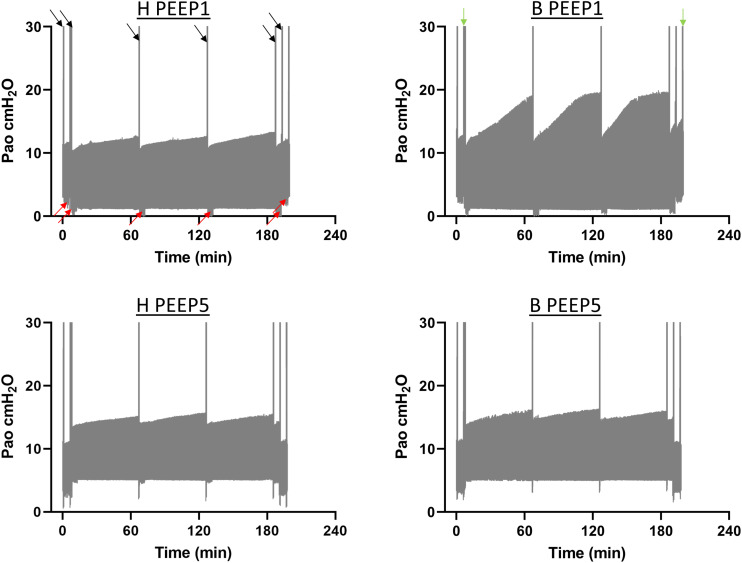
The airway opening pressure (Pao) at the expiratory limb of the rodent ventilator were continuously recorded during the mechanical ventilation in *n* = 3 lungs per experimental group. The graphs illustrate representative examples of the range of Pao for the different study groups. The lower limit represents the PEEP level while the upper limit corresponds to the peak inspiratory pressures. The black arrows indicate pressure increases during recruitment maneuvers (=deep inflations) which were followed by repetitive measurements of tissue elastance using the forced oscillation perturbation (FOT, red arrows). The green arrows indicate recording of pressure controlled pressure volume loops with a stepwise increase of Pao up to a maximum of 30 cmH_2_O (quasistatic PV-loops). Hence, the second bar is a combination of quasistatic PV-loops and deep inflations. The peak inspiratory pressures increased between the recruitment maneuvers in all study groups but did in none of the recorded datasets exceed 20 cmH_2_O.

**FIGURE 2:**
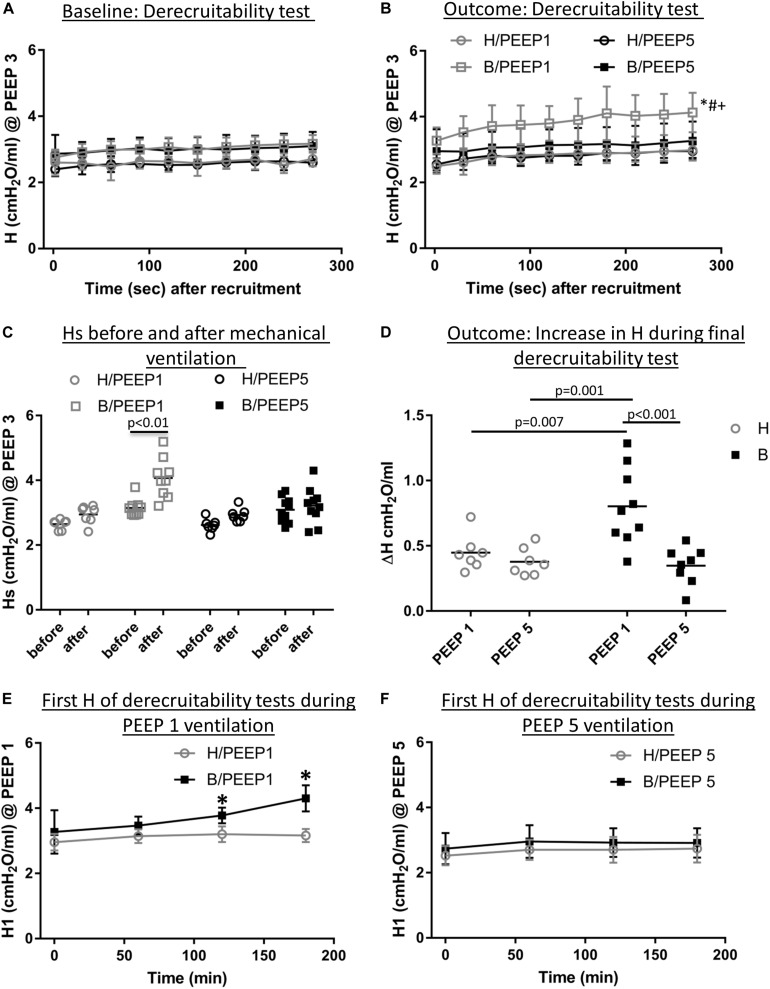
Lung mechanical data. **(A)** Derecruitability tests at baseline: Tissue Elastance (cmH_2_O/ml) was measured repetitively after first recruitment maneuver for 10 times before the onset of mechanical ventilation. H1 did not differ between study groups while Hs demonstrated significant Bleomycin effects. The data show that there was no difference present before subjects were assigned to the different ventilation protocols. Mean and standard deviation of at least seven independent measurements per group are given. **(B)** Shows derecruitability tests during PEEP = 3 cmH2O ventilation after 3 h of mechanical ventilation. Hs was significantly increased in B/PEEP1 compared to the other groups, including B/PEEP5. Mean and standard deviation of at least seven independent measurements per group are given. **(C)** Compares Hs data before and after mechanical ventilation in the different study groups. A statistical significant increase was only observed in B/PEEP1. **(D)** The increase in tissue Elastance (ΔH) was calculated from derecruitability tests after mechanical ventilation. Compared to the other groups, a significantly larger ΔH was observed in B/PEEP1. **(E)** The development of H1 is presented for H/PEEP1 and B/PEEP 1 during mechanical ventilation. Whereas H1 increased significantly (*p* < 0.05) in B/PEEP1, H1 remained stable in H/PEEP1. The same measurements are shown for H/PEEP5 and B/PEEP5 in **panel F**, where no increase can be identified in any of study groups. In **(E,F)** the mean and standard deviation of at least seven independent measurements per group are given. Statistics is based on two-way ANOVA followed by Bonferroni’s *post hoc* adjustment of the *p*-value in all graphs except for C where a dependent *t*-test was used. The level of statistical significance was *p* < 0.05. Hs: tissue elastance at steady state (mean of last 3 tissue elastance measurement of derecruitability test), H1: first tissue elastance after recruitment, ΔH = Hs–H1.

Continuous recording of the airway opening pressure (Pao) describes the time course of the peak inspiratory pressure (PIP) during mechanical ventilation. Representative examples of the recorded Pao data are illustrated in Figure 1. In general, in all recorded datasets the PIP increased between the derecruitability tests and could be reduced by a deep inflation (= recruitment manoeuvre). The increase in PIP between derecruitability tests was most pronounced in B/PEEP1. However, in none of the datasets did the peak inspiratory pressures exceed 20 cmH_2_O. Derecruitability tests were performed at PEEP = 3 cmH_2_O both before and after the 3-hour period of mechanical ventilation. Since the PEEP level has significant effects on tissue elastance (Knudsen et al., 2018), these measurements were necessary to obtain lung mechanical data under exactly the same conditions for comparison between study groups. The data of the 4 study groups during PEEP = 3 cmH_2_O ventilation are illustrated in Figure 2A (baseline) and Figure 2B (outcome after mechanical ventilation). H1 did not show any differences between study groups and the factor “Bleo” did not affect H1 at baseline according to the two-way ANOVA. However, the tissue elastance increased during the time course of the derecruitability test in particular in the bleomycin group. Hence, the level of tissue elastance values at the end of the derecruitability test (Hs), was slightly higher in the groups having been challenged with bleomycin (groups B/PEEP1 and B/PEEP5) compared to those which were healthy (groups H/PEEP1 and H/PEEP5) before starting either PEEP = 1 or 5 cmH_2_O ventilation. Accordingly, the two-way ANOVA demonstrated a significant effect of the factor “Bleo” on Hs during PEEP = 3 cmH_2_O ventilation (*p* < 0.01). No differences between the groups which were randomized to either PEEP1 or PEEP5 ventilation could be detected at baseline so that the study groups entered the different ventilation protocols with comparable lung mechanical properties.

After 3 h of mechanical ventilation the tissue elastance data in the B/PEEP1 group was significantly elevated above the other groups including B/PEEP5 as is shown in Figure 2B. With this regard, both bleomycin challenge (*p* = 0.001) as well as the PEEP level (*p* = 0.009) had a significant effect on Hs and these two factors demonstrated a significant interaction (*p* = 0.028). The study design also allowed within-group comparison of Hs before and after the 3 h period of mechanical ventilation. Using a student’s *t*-test for dependent measurements a highly significant increase in Hs was observed in the B/PEEP1 group but not in the other three groups B/PEEP5, H/PEEP1 and H/PEEP5 (Figure 2C). The increase of tissue elastance during the observational period after the recruitment manoeuvre, ΔH, was significantly increased in B/PEEP1 compared to the other three groups (Figure 2D) indicating increased progressive stiffening in B/PEEP1 after recruitment compared to the other groups. Derecruitability tests were also performed every 60 min during mechanical ventilation with PEEP = 1 or 5 cmH_2_O. Figures 2E,F illustrate the initial tissue elastance after recruitment manoeuvre, H1, as a function of time of mechanical ventilation during PEEP = 1 and 5 cmH_2_O ventilation. Changes in the first tissue elastance measurement after recruitment manoeuvre have been shown to correlate with the degree of alveolar epithelial injury based on quantitative ultrastructural data (Smith et al., 2017; Hamlington et al., 2018). During PEEP = 1 cmH_2_O ventilation the first tissue elastance after recruitment remained roughly stable in healthy lungs (H/PEEP1) but demonstrated a significant increase with time in the bleomycin challenged lungs (B/PEEP1) (Figure 2E, bleomycin effect *p* = 0.003, time effect *p* < 0.001, interaction < 0.001). Of note, both H/PEEP1 and B/PEEP1 started at the same level without any significant differences. On the other hand, during PEEP = 5 cmH_2_O both bleomycin challenged (B/PEEP5) as well as healthy lungs (H/PEEP5) did not show a temporal increase in H1. The level of H1 differed between PEEP = 1 cmH_2_O and PEEP = 5 cmH_2_O ventilation in both B and H and this is the effect of the increased onset pressure the FOT started (Knudsen et al., 2018).

Taken together, during and after the 3 h period of mechanical ventilation, there is a significant increase in tissue elastance in bleomycin challenged lungs ventilated with PEEP = 1 cmH_2_O. In the other study groups, tissue elastance remained comparably stable. In none of the study groups a relevant respiratory failure in terms of deoxygenation could be detected.

### Effects of Mechanical Ventilation on Lung Architecture

**FIGURE 3:**
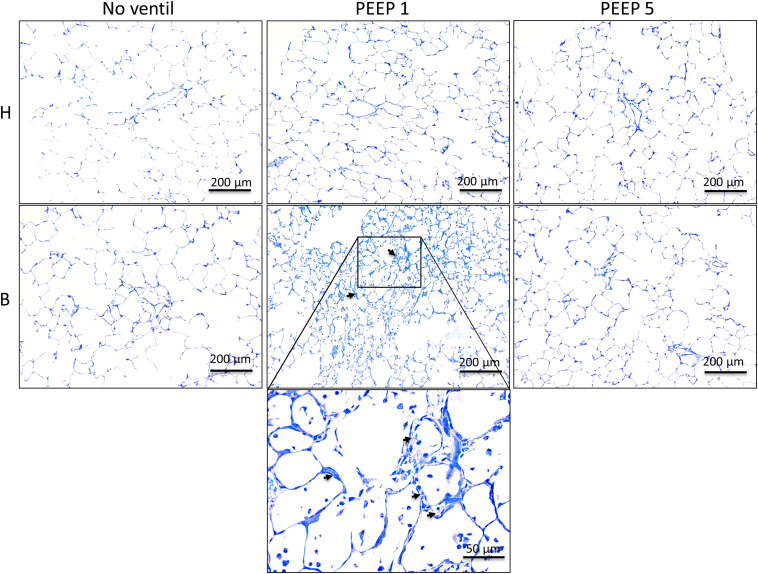
Representative toluidine blue stained light microscopic images are shown after airway instillation fixation at low magnification. Septal walls are slim and infiltrates are missing in H with and without mechanical ventilation. Inflammatory cells and thickened inter-alveolar septal walls (arrows) can be seen in B/PEEP1 but not in B/PEEP5.

**FIGURE 4:**
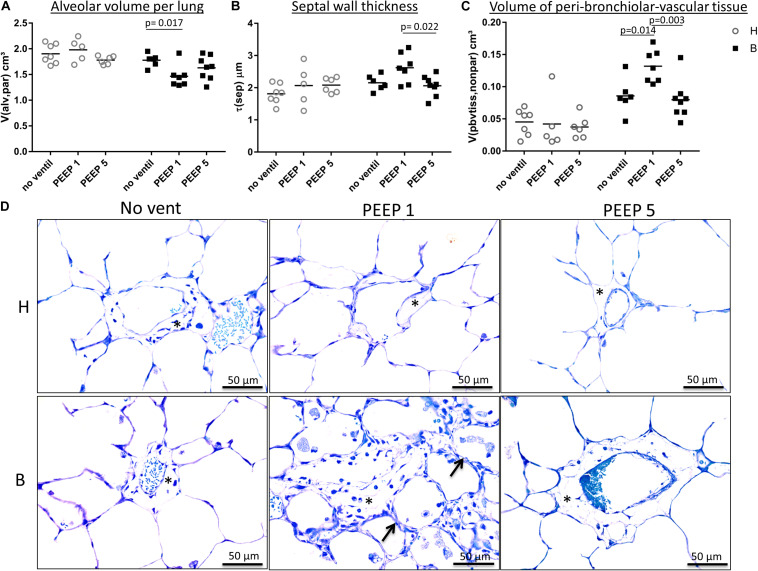
Light microscopical stereological data and interstitial edema. **(A)** The absolute volume of alveolar airspaces (V(alv,par) was reduced in group B compared to group H. Within group B data were lowest in B/PEEP1. **(B)** Within group B a significant effect of ventilation on arithmetic mean septal wall thickness was observed resulting in an increase in B/PEEP1 compared to B/PEEP5. **(C)** Bleomycin pre-treatment resulted in an increase in the volume of peri-bronchiolar vascular connective tissue (V(pbvtis,nonpar)). Within group B a significant ventilation effect could be observed resulting in largest values in B/PEEP1 compared to both B/no ventil as well as B/PEEP5. Statistics were made with the use of two-way ANOVA and Bonferroni’s multiple comparisons. **(D)** High magnification, representative light microscopic images after airway instillation fixation are shown, stained with toluidine blue. Healthy lungs show only marginal peri-bronchiolar-vascular connective tissue (asterisks), whereas B/PEEP1 reveals cellular and edema influx into the non-parenchymal connective tissue compared with all other groups, which is further confirmed by our quantitative measurements in 3C. Also, thickened septal walls were observed in B/PEEP1 (arrows).

**FIGURE 5:**
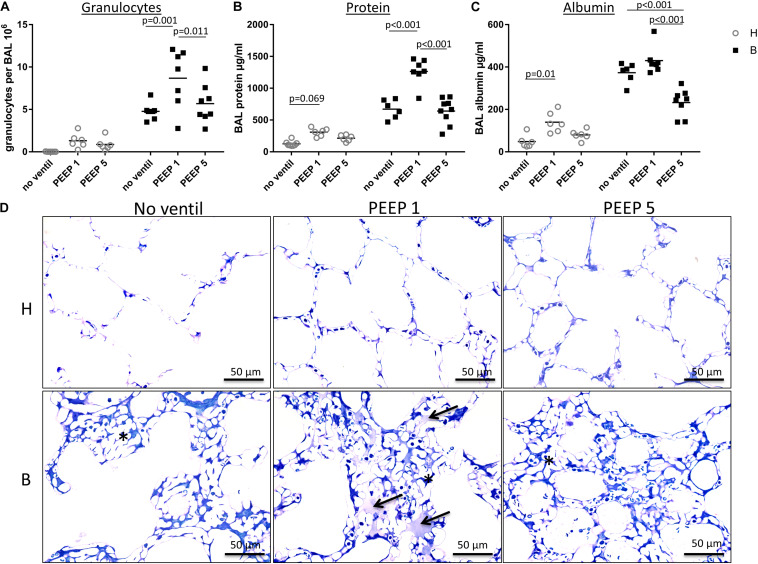
BAL data and intra-alveolar edema. **(A)** In this figure, the amount of neutrophil granulocytes within the BAL is presented for each group and demonstrates an enormous influx of neutrophil granulocytes into the alveolar space in group B/PEEP1. This influx is statistically significantly higher compared to B/PEEP5 and B/no ventil. **(B)** The BAL protein concentration in μg/ml is shown. For group B/PEEP1, the protein content is significantly elevated versus B/PEEP5 (*p* < 0.001) and B (*p* < 0.001). Furthermore, initially healthy lungs demonstrate a trend for an increased amount of BAL protein level after ventilation with PEEP = 1 cmH_2_O (H/PEEP1). **(C)** The concentrations of BAL albumin in μg/ml show a slightly different distribution than the protein measurements but highest albumin concentration can be identified in group B/PEEP1 which differs significantly from B/PEEP5 but not from B/no ventil. Of note, the albumin level is significantly lower in B/PEEP5 compared to B/no ventil. There is a significant difference between H and H/PEEP1 (*p* = 0.01). **(D)** Light microscopic images after vascular perfusion fixation at end-inspiratory pressure during PEEP = 1 cmH_2_O ventilation. Sections were stained with toluidine blue. Microatelectases or alveolar edema are absent in healthy lungs. All Bleomycin-treated lungs demonstrate microatelectatic regions at end-inspiratory status (asterisk). In lungs from group B/PEEP1, alveolar edema fluid in adjacency to microatelectasis can be observed (arrows). Intraalveolar edema is hardly visible in B/PEEP5.

Left lungs were instillation fixed and available for qualitative and quantitative structural assessments. Additional pairs of lungs per study group were fixed by vascular perfusion at end-inspiration to describe microatelectases and intra-alveolar edema fluid at light microscopic level. These pathological alterations are usually not preserved in lungs fixed by airway instillation (Hsia et al., 2010; Lutz et al., 2015). Representative images of instillation fixed lungs are illustrated in Figures 3, 4D while images of perfusion fixed lungs are shown in Figure 5D. The healthy lungs, independent of ventilation pattern, did not show any evidence of increased numbers of inflammatory cells within the airspaces or the peri-bronchiolar-vascular connective tissue compartment (Figure 3). The interalveolar septa were not thickened and the distal airspaces were open. The bleomycin-challenged lungs which were either not mechanically ventilated or ventilated with PEEP 5 cmH_2_O looked very similar to the healthy lungs (Figure 3). In contrast, PEEP = 1 cmH_2_O ventilation of bleomycin-challenged lungs was characterized by more inflammatory cells within the distal airspaces as well as in the peri-bronchiolar-vascular connective tissue compartment. In addition, in some areas of lung parenchyma, the interalveolar septal walls appeared to be thickened (Figure 3).

Instillation fixed lungs were subjected to stereological analyses at light microscopic level. These data are presented in Table 1 and illustrated in Figure 4. The total lung volume (V(lung)) was not affected by bleomycin or the mechanical ventilation pattern. On the contrary, the total volume of lung parenchyma (V(par,lung)) as well as non-parenchyma (V(nonpar,lung)) per lung demonstrated significant bleomycin effects: V(par,lung) was reduced while V(nonpar,lung) was increased. Within the bleomycin-treated lungs, the Bonferroni *post hoc* comparison demonstrated significantly lower V(par,lung) in B/PEEP1 compared to B/No ventil (*p* = 0.047). On the other hand V(nonpar,lung) was largest in B/PEEP1 but no significant differences from B or B/PEEP5 were identified. The parenchyma was divided into the compartments of alveolar airspaces (V(alv,par)), ductal airspaces (V(duct,par)) and interalveolar septa (V(sep,par)). With regard to V(alv,par) a significant effect of the factor “bleomycin treatment” could be identified resulting in a decrease. Within the bleomycin challenged groups a significant reduction of V(alv,par) could be identified in B/PEEP1 compared to B/No ventil while no significant difference existed between B/PEEP5 and B/No ventil or B/PEEP1 (Figure 4A). In a further step, the alveolar surface area (S(alv,par)) within the lung was determined and this parameter was affected by both the bleomycin treatment and the mechanical ventilation pattern. S(alv,par) was reduced by bleomycin pre-treatment and mechanical ventilation resulted in an additional reduction, which was most prominent in B/PEEP1. Compared to B/No ventil, S(alv,par) was significantly reduced in B/PEEP1 (*p* = 0.014) but also in B/PEEP5 (*p* = 0.033). The arithmetic mean thickness of interalveolar septa [t(sep)] was calculated as a volume-to-surface ratio and data are illustrated in Figure 4B. While the bleomycin-challenge itself had a significant effect on this parameter, the Bonferroni test revealed that t(sep) was significantly higher in B/PEEP1 compared to B/PEEP5 (*p* = 0.022), indicating a ventilation effect on this parameter.

**TABLE 1:** Summarized stereological data are given as mean (standard deviation).

Parameter	H			B			Two-way ANOVA		
	
	No ventil	PEEP1	PEEP5	No ventil	PEEP1	PEEP5	Bleo	Vent	inter
V(lung) cm^3^	2.72 (0.24)	2.80 (0.29)	2.71 (0.15)	2.74 (0.17)	2.52 (0.29)	2.55 (0.27)	0.10	0.56	0.313
V(par,lung) cm^3^	2.47 (0.27)	2.58 (0.28)	2.53 (0.12)	2.49 (0.15)	2.16 (0.28)*	2.29 (0.23)	0.006	0.53	0.068
V(nonpar,lung) cm^3^	0.25 (0.1)	0.22 (0.1)	0.17 (0.04)	0.26 (0.05)	0.36 (0.08)	0.26 (0.1)	0.005	0.12	0.128
V(alv,par) cm^3^	1.90 (0.19)	1.98 (0.22)	1.78 (0.08)	1.78 (0.12)	1.47 (0.22)*	1.63 (0.23)	<0.001	0.15	0.027
V(duct,par) cm^3^	0.44 (0.08)	0.47 (0.06)	0.63 (0.07)*^#^	0.58 (0.07)	0.56 (0.1)	0.54 (0.09)	0.099	0.033	0.003
V(sep,par) cm^3^	0.12 (0.02)	0.13 (0.04)	0.12 (0.02)	0.13 (0.01)	0.14 (0.03)	0.11 (0.02)	0.779	0.151	0.379
S(alv,par) cm^2^	1299 (79)	1306 (120)	1191 (62)	1232 (81)	1066 (126)*	1089 (104)*	<0.001	0.009	0.089
τ(sep) μm	1.81 (0.3)	2.07 (0.63)	2.08 (0.24)	2.15 (0.24)	2.62 (0.46)	2.06 (0.32)^#^	0.022	0.062	0.169
V(airway,nonpar) mm^3^	131 (74)	128 (32)	126 (28)	127 (22)	173 (63)	143 (48)	0.247	0.559	0.483
V(ves,nonpar) mm^3^	58.4 (31.7)	45.3 (46.1)	20.7 (8.7)*	42.9 (8.4)	56.0 (6.8)	40.1 (22.2)	0.536	0.057	0.17
V(pbvtis,nonpar) mm^3^	45.2 (20.4)	42.0 (42.0)	37.5 (17.6)	85.8 (27.3)	131.9 (25.3)*	79.9 (30.2)^#^	<0.001	0.041	0.058
S(AE1_intact,par) cm^2^	918 (274)	792 (103)	820 (192)	421 (115)	263 (92)	504 (177)^#^	<0.001	0.108	0.275
S(AE1_injure,par) cm^2^	60 (36)	128 (93)	201 (122)	305 (79)	455 (187)*	204 (111)^#^	<0.001	0.017	0.012
S(AE2_intact,par) cm^2^	57.4 (39.4)	41 (24)	94.2 (47.5)	24.1 (25.6)	40.6 (31.3)	125 (180)	0.951	0.083	0.647
S(AE2_injure,par) cm^2^	0 (0)	1.0 (2.3)	3.0 (3.5)	6.7 (6.0)	12.6 (8.7)	11.6 (13.2)	0.001	0.389	0.795

*A two-way ANOVA on ranks was performed to take the factors of treatment (bleomycin effects, B vs. H) and ventilation (no ventil vs. PEEP1 vs. PEEP5) as well as their interaction (inter) into consideration. Within each treatment group (B and H) an adjustment of the p-level for multiple testing using the Bonferroni *post hoc* test was added. Within each group (B and H) statistical significant differences of ventilation effects are indicated as follows: ^∗^*p* < 0.05 vs. no ventil; ^#^*p* < 0.05 vs. PEEP1. H, healthy; B, bleomycin pre-treatment; Vent, ventilation pattern; V, volume; S, surface area; τ, arithmetic mean thickness; par, lung parenchyma; nonpar, non-parenchyma; alv, alveolar airspace; duct, ductal airspace; sep, interalveolar septa; airway, conducting airway; ves, blood vessel; pbvtis, peri-bronchiolar-vascular connective tissue; AE1, alveolar epithelial type 1 cell; AE2, alveolar epithelial type 2 cell.*

The different compartments of non-parenchyma were categorized into the total volumes of vessels (V(ves,nonpar)), conducting airways (V(airway,nonpar)) and peri-bronchiolo-vascular connective tissue (V(pbvtis,nonpar)). V(ves,nonpar) and V(airway,nonpar) did not show any significant differences. However, V(pbvtis,nonpar) was clearly effected by both the bleomycin-challenge and the mechanical ventilation pattern (Figure 4C). Prior to mechanical ventilation, bleomycin pre-treatment (group B/No ventil) resulted in a near doubling of V(pbvtiss,nonpar) compared to healthy controls (group H/No ventil). Mechanical ventilation with PEEP1 in addition to bleomycin challenge (B/PEEP1) resulted in a further increase so that V(pbvtiss,nonpar) became significantly larger compared to B/No ventil (*p* = 0.015) but also compared to B/PEEP5 (*p* = 0.003). A closer look at the peri-bronchiolo-vascular connective tissue compartment demonstrated an increased infiltration with inflammatory cells but also very prominent, enlarged lymphatic vessels in B/PEEP1 in comparison to the other study groups (Figure 4D). The adjacent interalveolar septa of infiltrated and thickened connective tissue cuffs around airways and vessels appeared to be thickened (Figure 4D). In healthy lungs, mechanical ventilation did not have any effects on the peri-bronchiolo-vascular connective tissue compartment.

Taken together, low PEEP mechanical ventilation of bleomycin pre-treated lungs resulted in an additional decrease in the volume of alveolar airspaces and surface area of alveoli. The interalveolar septa were thickest and the total volume of peri-bronchiolo-vascular connective tissue largest in B/PEEP1.

### BAL Measurements

The right lungs were subjected to broncho-alveolar lavage. Altogether 12 ml of 0.9% NaCl solution were instilled per lung and between 10 and 11 ml were recovered. Figures 5A–C illustrates the data obtained from the BAL measurements. While the total numbers of alveolar macrophages and lymphocytes per BAL did not differ between the study groups (data not shown), the number of neutrophilic granulocytes was markedly increased and was influenced by both the bleomycin pre-treatment (*p* < 0.001) and the mechanical ventilation pattern (*p* = 0.003). No significant interaction between the factors bleomycin pre-treatment and PEEP level were observed (*p* = 0.131). Within the healthy lungs, however, the mechanical ventilation did not show statistical significant differences. In bleomycin pre-treated lungs on the other hand highest numbers of neutrophilic granulocytes were found in B/PEEP1 and this was statistically significant compared to both B/No ventil and B/PEEP5 (Figure 5A). Hence, PEEP 1 ventilation but not PEEP 5 ventilation resulted in an additional increase in neutrophilic granulocytes after bleomycin pre-treatment.

The levels of protein (Figure 5B) and albumin (Figure 5C) were measured within the BAL as surrogate markers for leakage of the blood-gas barrier. Bleomycin pre-treatment (*p* ≤ 0.001) and PEEP level (*p* < 0.001) had significant effects on protein and albumin levels and also demonstrated relevant interactions with regard to these parameters (*p* < 0.001). Bleomycin challenge without ventilation significantly increased protein (*p* < 0.001) and albumin (*p* < 0.001) levels. Investigation of the effects of the ventilation pattern within the bleomycin challenged group showed elevated BAL protein in B/PEEP1 compared to both B/No ventil (*p* < 0.001) and B/PEEP5 (*p* < 0.001). Within the healthy lungs BAL protein trended higher in H/PEEP1 compared to H (*p* = 0.069). The BAL albumin level behaved differently from the protein level (Figure 5C). PEEP 1 ventilation after bleomycin challenge did not result in an additional increase in albumin level so that there are no statistical differences between B and B/PEEP1. On the other hand, B/PEEP5 had significant lower albumin levels compared to B (*p* < 0.001) and B/PEEP1 (*p* < 0.001). Within initially healthy lungs, PEEP = 1 cmH_2_O ventilation resulted in a significant increase in albumin levels (*p* = 0.01), a finding which could not be observed after PEEP = 5 cmH_2_O ventilation. Measurements of IL-6 were performed in the BAL since this parameter has been shown to correlate with the outcome in an animal model of VILI (Szabari et al., 2019). While in healthy control, the level of IL-6 was underneath the detection level, mechanical ventilation resulted in an increase which was more pronounced in H/PEEP1 [mean = 522 (SD = 238) pg/ml] than in H/PEEP5 [mean = 179 (SD = 115) pg/ml]. Bleomycin challenge resulted in measureable IL-6 levels in BAL [mean = 123 (SD = 54) pg/ml] which increased in B/PEEP1 [mean = 779 (SD = 246) pg/ml] and B/PEEP5 [mean = 959 (SD = 739) pg/ml] groups to a similar degree.

Taken together, PEEP1 but not PEEP5 ventilation of bleomycin pre-injured lungs aggravated markers of lung injury including the number of BAL granulocytes and BAL protein level. From previous studies it is known that the volume of edema fluid one day after bleomycin-induced lung injury is increased based on electron microscopic assessments but cannot be detected at light microscopic level since the protein rich edema covering the alveolar walls is too thin and beyond light microscopic resolution (Knudsen et al., 2018). In the present study we investigated lungs at light microscopic level which were fixed during PEEP = 1 cmH_2_O ventilation at an end-inspiratory arrest by vascular perfusion fixation (Figure 5D). The goal was to identify microatelectases and intraalveolar edema fluid. Healthy lungs, independent of mechanical ventilation did not show any signs of microatelectases or intraalveolar edema. On the other hand, all bleomycin pre-treated lungs during PEEP = 1 cmH_2_O ventilation demonstrated end-inspiratory microatelectases as a common finding. No intraalveolar edema fluid could be detected in lungs after PEEP5 ventilation or those which were not mechanically ventilated. After PEEP1 ventilation, however, there were traces of intraalveolar edema fluid visible in some regions at light microscopic level and these were in general co-located with areas of microatelectases. Hence, mechanical ventilation of bleomycin pre-injured lungs with PEEP1 but not with PEEP5 seems to aggravate intraalveolar edema fluid so that it becomes clearly detectable at light microscopic level.

### Injury of the Blood-Gas Barrier

**FIGURE 6:**
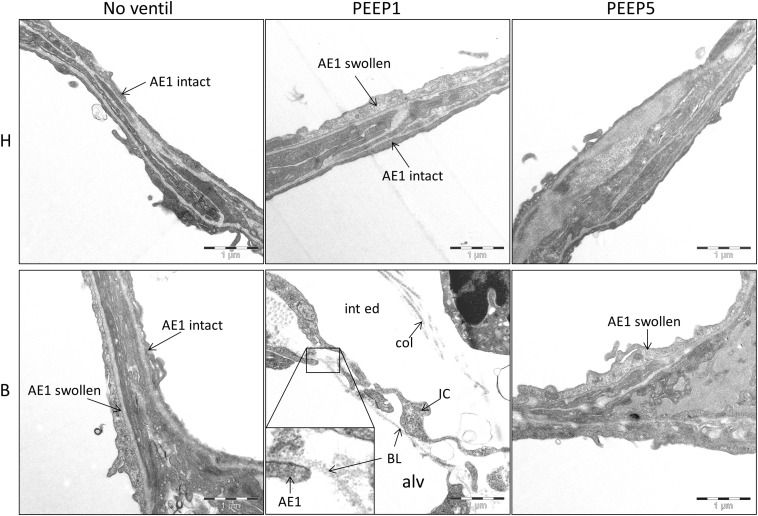
Injury of alveolar epithelial cells. Representative electron-microscopic images are shown at primary magnification of 11,000×. There is evidence of epithelial injury, like swollen AE1 cells, in most of the experimental groups, except H/No ventil. Regarding B/PEEP1, the injury of the blood-gas barrier was severe, since complete denudations of the epithelial basal lamina (BL) was observed in particular in areas characterized by a widening of the septal walls due to interstitial fluid accumulation (int ed). col, collagen fibrils; IC, interstitial cell; alv, alveolar airspace.

**FIGURE 7:**
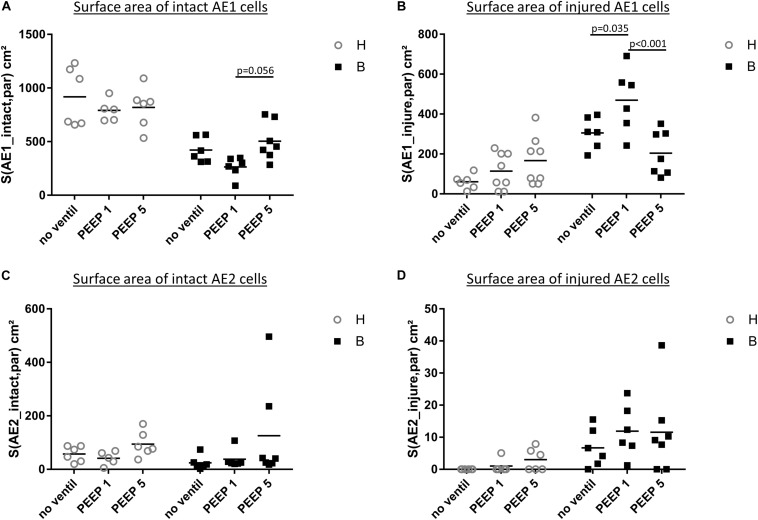
Stereological data of alveolar epithelial injury. **(A)** The surface area of basal lamina covered by intact (healthy) AE1 cells (S(AE1_intact,par) was larger in group H compared to group B. Within group B a tendency for reduced values were found in B/PEEP1 compared to B/PEEP5. **(B)** The surface area of basal lamina covered by injured AE1 cells S(AE1_injured,par) increases in group B compared to group H. This was most pronounced in B/PEEP1 where S(AE1_injured,par) was significantly larger compared to B/no ventil or B/PEEP5. No significant effect of mechanical ventilation was found within H. The surface area of basal lamina covered by healthy **(C)** or injured AE2 cells S(AE2_intact,par) **(D)** demonstrates a Bleomycin but no ventilation effect on S(AE2_intact,par).

Microatelectases have been suggested to function as stress concentrators and might induce volutrauma in neighboring interalveolar septa during mechanical ventilation via alveolar interdependence. This mechanism would result in ruptures of the cellular components of the blood-gas barrier (Cong et al., 2017) followed by leakage. Accordingly, the blood-gas barrier was investigated for ultrastructural signs of injury in the present study. Representative micrographs are provided in Figure 6. Signs of injury of the alveolar epithelial type 1 (AE1) cells were found in all ventilated lungs and, in addition, in unventilated bleomycin treated lungs. Except for group B/PEEP1 the injury was characterized by a swelling of AE1 cells combined with a clearing of their cytoplasmic ground substance. In group B/No ventil this was the typical ultrastructural presentation of the injury of the blood-gas barrier and more severe signs of injury were hardly present, reproducing previous observations in this animal model (Lutz et al., 2015). In B/PEEP1, however, complete denudations of the epithelial basal lamina were observed quite frequently (Figure 6). This was accompanied by thickening of the interalveolar septa and a divergence of the formed extracellular components such as the collagen fibrils due to interstitial fluid accumulation. The degree of injured epithelial cells was quantified by determining the total surface area of the basal lamina covered by healthy or injured AE1 cells as well as AE2 cells (Figure 7). Bleomycin and the ventilation pattern had both a significant effect on the surface area covered by injured AE1 cells (Table 1). While in bleomycin pre-injured lungs the PEEP = 1 cmH_2_O ventilation resulted in an additional, although not significant (*p* = 0.056), decrease in surface area covered by intact AE1 cells (Figure 7A) combined with an increase in surface area covered by injured AE1 cells (Figure 7B), this was not the case with PEEP = 5 cmH_2_O ventilation where no difference from non-ventilated bleomycin lungs were observed. In the healthy lungs, both PEEP levels resulted in a slight, although statistically not significant increase in the surface area of injured AE1 cells. In general, the effect of bleomycin and/or mechanical ventilation on ultrastructural AE2 cell injury markers was much less pronounced compared to AE1 cells. In all study groups the ratio of injured AE2 cells to intact AE2 cells was much smaller compared to the ratio of injured AE1 cells and intact AE1 cells. Neither bleomycin nor mechanical ventilation demonstrated an effect on the surface area covered by intact AE2 cells (Figure 7C). On the other hand, bleomycin, but not the ventilation pattern, demonstrated an effect on the surface area covered by injured AE2 cells (Figure 7D). Within the group of bleomycin challenged lungs no significant differences in AE2 injury attributable to ventilation pattern were observed.

Taken together, mechanical ventilation of a bleomycin pre-injured lung with a low PEEP increased the surface area of epithelial basal lamina covered by of abnormal appearing AE1 cells. This behavior could not be observed during mechanical ventilation with PEEP = 5 cmH_2_O.

### Cellular Stress Markers of Alveolar Epithelial Cells

**FIGURE 8:**
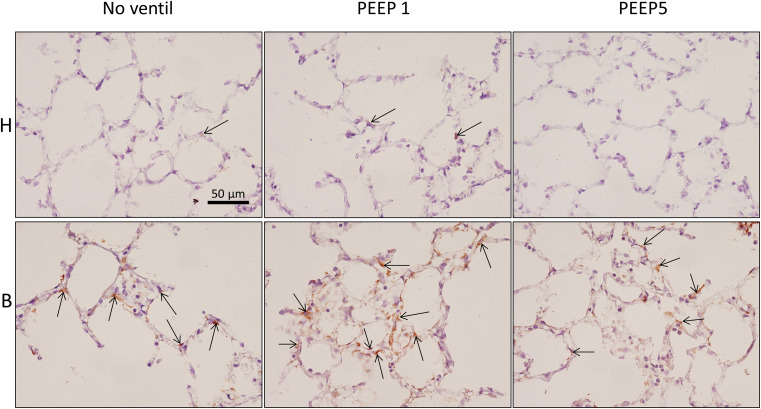
Endoplasmic reticulum stress. p-Perk positive cells (arrows), stained by immunohistochemistry, were found in all experimental groups but most profiles of p-Perk positive cells were present in B. There was no clear effect of mechanical ventilation visible.

**FIGURE 9:**
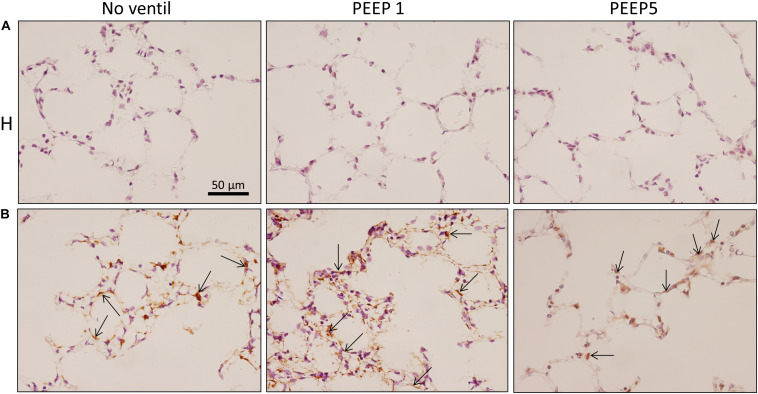
Integrated stress response. Immunohistochemistry of p-EIF-2α is shown (arrows). While no staining was seen in H, bleomycin alone resulted in a positive labeling of cells in septal walls and this appears to be aggravated by mechanical ventilation with PEEP = 1 cmH_2_O in some regions. Qualitatively, p-EIF-2α labeled cells were predominantly found in adjacency to microatelectatic regions.

*In vitro* models of increased epithelial stretch demonstrated that the integrated stress response is activated by mechanical stress of primary alveolar epithelial cells (Dolinay et al., 2017, 2018). Therefore, we performed immunohistochemical staining of p-Perk and p-EIF-2α in four sections per experimental group. Staining with isotype control antibody (rabbit IgG) did not show any evidence of unspecific binding of the primary antibodies used against p-Perk or p-EIF-2α (Supplementary Figure S1). Regarding p-Perk, positive cells could occasionally be detected in healthy lungs independent of whether or not the lungs were mechanically ventilated. These positive cells were predominantly found within the septal wall. After bleomycin and mechanical ventilation, alveolar epithelial p-Perk expression demonstrated a marked increase (Figure 8). p-EIF-2α was not detectable in healthy lungs before or after mechanical ventilation. After bleomycin instillation, however, alveolar epithelial cells were positive for p-EIF-2α. Mechanical ventilation of bleomycin-treated lungs appeared to increase the fraction of p-EIF-2α positive cells particularly in and in close proximity to microatelectases (Figure 9). For quantification, the fraction of the alveolar surface area which stained positive for p-EIF-2α was determined by means of intersection counting. Since healthy lungs did not show any positive cell this investigation was only performed in bleomycin challenged experimental groups. The mean and standard deviation of the fraction of alveolar surface area covered by p-EIF-2α positive cells of *n* = 4 sections per study group were 0.23 (0.04), 0.35 (0.07), and 0.21 (0.03) for B/No ventil, B/PEEP1 and B/PEEP5, respectively. In B/PEEP1 a trend for a significantly larger surface fraction was found compared to B/No ventil (*p* = 0.055).

In summary, mechanical ventilation with PEEP1 increased the surface fraction of p-EIF-2α positive epithelial cells by trend, a finding which correlated with the occurrence of p-Perk positive epithelial cells.

### Correlation Analyses

**FIGURE 10:**
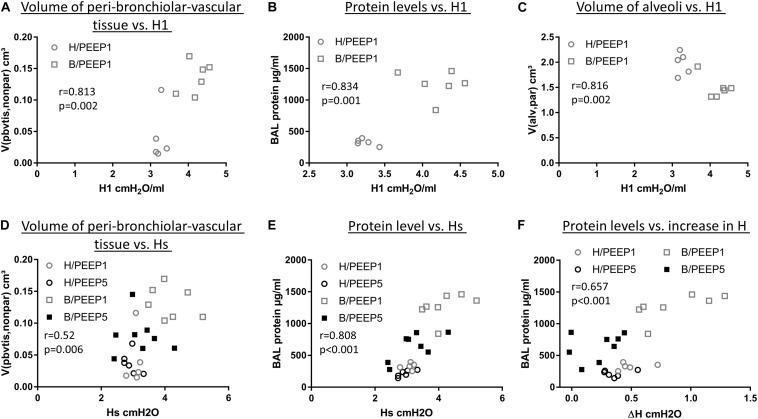
Correlation with lung mechanical data. H1 in the last derecruitability test performed after 3 h of mechanical ventilation with PEEP = 1 cmH_2_O demonstrated strong positive correlations with the volume of peri-bronchiolo-vascular connective tissue **(A)** and BAL protein content **(B)** and a negative correlation with the absolute volume of alveolar airspaces in lung parenchyma (V(alv,par)) **(C)**. Hs, referred to as the mean of the last three measurements of H during the derecruitability test at PEEP = 3 cmH_2_O ventilation performed after the 3 h period of mechanical ventilation, was tested for correlation to the volume of peri-bronchiolo-vascular connective tissue (V(pbvtis,nonpar) **(D)** and BAL protein concentration **(E)** and both parameters demonstrated a strong correlation with Hs. Regarding the increase in tissue elastance during the derecruitability test at PEEP = 3 cm H_2_O ventilation performed after the 3 h period of mechanical ventilation, ΔH, the BAL protein level showed the strongest correlation **(F)**. Hs: tissue elastance at steady state, H1: first tissue elastance after recruitment, ΔH = Hs–H1.

During mechanical ventilation with PEEP = 1 cmH_2_O, H1 increased with time in the bleomycin pre-treated lungs while it remained stable in the healthy lung. With the goal to investigate which abnormalities in lung structure and BAL measurements were best linked with this increase in H1, correlation analyses were performed. The total volume of peri-bronchiolar-vascular connective tissue per lung (V(pbvtis,nonpar), Figure 10A), protein and albumin levels in BAL (Figure 10B), the surface area of basal lamina covered by injured alveolar epithelial cell as well the volume of alveolar airspaces (Figure 10C) demonstrated highly significant (*p* < 0.01) correlations with H1 measured at PEEP = 1 cmH_2_O.

Hs measured at the conclusion of ventilation during PEEP = 3 cmH2O ventilation demonstrated significant correlations with arithmetic mean thickness of septal walls [t(sep), *r* = 0.575, *p* < 0.01)] and peri-bronchiolar-vascular connective tissue (V(pbvtis,par), Figure 10D) but also the mean volume of alveolar airspaces (V(alv,par), *r* = 0.619, *p* < 0.01). The most statistical correlation with Hs, however, could be established for BAL protein (Figure 10E) and albumin level (*r* = 0.788, *p* < 0.01). The increase in tissue elastance during 4.5 min of low tidal volume, PEEP = 3 cmH_2_O ventilation following a recruitment manoeuvre (ΔH) demonstrated statistically significant correlations with BAL protein (Figure 10F) and BAL albumin (*r* = 0.616, *p* < 0.01) levels only while correlations with structural data were, at best, moderate. The BAL protein or albumin level were highly correlated to the total volume of peri-bronchiolar-vascular connective tissue as well as to the surface area of the epithelial basal lamina covered by injured epithelial type I cells (data not shown).

## Discussion

Abnormalities of alveolar micromechanics may not always be reflected in measurements at the organ scale such as arterial blood oxygenation or lung mechanical properties (Knudsen et al., 2018; Grune et al., 2019). On the other hand, it has been shown that abnormal alveolar micromechanics can aggravate acute lung injury during mechanical ventilation by atelectrauma and volutrauma (Knudsen and Ochs, 2018). The mechanisms of atelectrauma and volutrauma are linked with each other via alveolar interdependence. Derecruited alveoli act as stress concentrators and could increase dynamic strain and therefore micromechanical volutrauma in adjacent, open alveoli (Mead et al., 1970; Makiyama et al., 2014; Albert et al., 2019). In healthy lungs both atelectrauma (e.g., a PEEP of 0 cmH_2_O) and volutrauma (e.g., high tidal volume of 30 ml/kg bodyweight) are required in combination to produce VILI in mice (Seah et al., 2011).

In the present study we tested the hypothesis whether hidden microatelectases increase VILI susceptibility during mechanical ventilation that does not harm healthy lungs. Such microatelectases have been shown to occur at end-expiratory airway pressures below 5 cmH_2_O in the early stage of bleomycin-induced lung injury (Knudsen et al., 2018). We termed these microatelectases one day after bleomycin instillation as “hidden” since oxygenation and tissue elastance, which represent typically available organ scale measurements during mechanical ventilation, were virtually unchanged compared to healthy controls (Knudsen et al., 2018). During mechanical ventilation of bleomycin challenged lungs with PEEP = 1 cmH_2_O the peak inspiratory pressure was at the very beginning within the range found in healthy lungs. However, with time the peak inspiratory pressure demonstrated a considerable and progressive increase in the bleomycin pre-treated lungs (Figure 1) so that the pre-existing injury became manifest at the organ level. The increase in peak inspiratory pressure could be reduced by recruitment manoeuvres and might therefore reflect the occurrence of progressive but still recruitable atelectasis during low PEEP mechanical ventilation (Allen et al., 2005).

In the current study we demonstrate that the presence of microatelectases during mechanical ventilation for 3 h is linked with increased interstitial edema (Figures 4B,C), pulmonary inflammation (Figure 5A), protein levels in BAL (Figure 5B), and (based on ultrastructural criteria) injury of the alveolar epithelial cells (Figures 6, 7). This ventilation-induced exacerbation of occult injury is reflected in organ-scale lung mechanics as shown in tissue elastance during and after the 3 h period of mechanical ventilation (Figure 2). During mechanical ventilation with PEEP = 1 cmH_2_O in presence of microatelectases (B/PEEP1) the first tissue elastance measured 2 s after a recruitment manoeuvre (H1) is characterized by a progressive increase with ventilation time (Figure 2E). This behavior was not observed in the lungs where we have previously not observed any microatelectases such as H/PEEP1 and B/PEEP5. Since H1 was measured immediately after recruitment it can be considered as a parameter reflecting lung mechanical properties at highest degree of recruitment and therefore components of lung injury which are not responsive to deep inflations (Massa et al., 2008; Smith et al., 2017). The increase in H1 during PEEP = 1 cmH_2_O ventilation was highly correlated with structural parameters reflecting interstitial edema such as the volume of peri-bronchiolar vascular connective tissue (Figure 10A).

In addition, markers reflecting pulmonary inflammation (the number of neutrophilic granulocytes per BAL) or leakage of protein into the airspace (protein and albumin levels) were highly correlated with H1 during PEEP = 1 cmH_2_O ventilation. The BAL data indicate progressive, protein-rich alveolar edema formation in B/PEEP1 and this is supported by qualitative light microscopical observations in lungs fixed by vascular perfusion at end-expiratory airway opening pressure of 1 cmH_2_O that show interposing alveolar edema within areas of microatelectases (Figure 5D). Since it has been previously shown that protein-rich alveolar fluid accumulation in the context of high surface tension reduces the recruitability of distal airspaces (Rühl et al., 2019) it can be speculated that in the present study the progressive increase in H1 with time reflects reduced alveolar recruitment. This can be a consequence of altered alveolar lining fluid rheology and/or progressive inactivation of pulmonary surfactant by plasma proteins (Seeger et al., 1993). In addition, increased dynamic strain of alveoli adjacent to microatelectases has also the potential to inactivate surfactant, e.g., by increased conversation from biophysically active large aggregates to inactive small aggregates (Veldhuizen et al., 1996; Albert, 2012). The increased dynamic alveolar strain might result in ruptures of the surfactant layer giving plasma proteins the opportunity to compete with the surfactant at the air-liquid interface ending up in a further destabilization of distal airspaces (Lopez-Rodriguez and Pérez-Gil, 2014).

Of note, the protein and albumin levels in the BAL fluid did not follow the same trend after mechanical ventilation of bleomycin challenged lungs. While PEEP = 1 cmH_2_O ventilation increased the protein level (Figure 5B) the albumin level remained roughly stable (Figure 5C). The protein level (but not the albumin level) correlated with the surface area of the epithelial basal lamina covered by injured type I alveolar epithelial cells (Figure 6B). The increase in BAL protein in B/PEEP1 can therefore hardly be explained by leakage of albumin from the blood into the alveolar space. Based on the ultrastructural findings the endothelial cells were unaffected by the mechanical ventilation so that the barrier function on the blood side seemed to be still intact and prevented an additional leakage of albumin into the airspace. Since the protein level correlates with ultrastructural signs of injury of alveolar epithelial cells leakage of intracellular proteins into alveolar space in combination with increased inflammation appears to be an important source for protein accumulation after PEEP 1 ventilation of bleomycin pre-injured lungs.

The largest increase in tissue elastance over the observational period of 4.5 min after a recruitment manoeuvre was found in the group B/PEEP1 (ΔH, Figure 2D). This behavior can be the consequence of an increased time-dependent derecruitment of distal airspaces (Massa et al., 2008; Smith et al., 2012) and therefore, in the context of the present study, a result of increased alveolar instability due to surfactant inactivation. The increase in ΔH was highly correlated to the albumin and protein levels in the BAL (Figure 10E), an observation which is in line with previous observations in a VILI mouse model (Smith et al., 2017). As opposed to Hs, ΔH demonstrated at best moderate correlations (e.g., *r* < 0.5) to structural data and this could be a consequence of the fact that ΔH reflects the dynamic, time-dependent alterations which have their foundation in the properties of the air-liquid interface, e.g., high surface tension or redistribution of alveolar liquid which is dependent on alveolar protein content but not represented in the stereological data. Hs, e.g., the tissue elastance under steady state conditions after recruitment manoeuvre, on the other hand is dependent on both the mechanical properties of the inter-alveolar septa (Knudsen et al., 2015) and the characteristics of the air-liquid interface (Mead, 1961).

In the current study, quantitative structural data were generated in lungs which were fixed by airway instillation at a hydrostatic pressure of 25 cmH_2_O. In these liquid-filled lungs the air-liquid interface has been abolished so that the effects of surface tension on lung structure, such as microatelectases or the location of intra-alveolar edema, cannot be investigated (Gil et al., 1979; Wilson and Bachofen, 1982). The fact that the volume of alveolar airspaces as well as the surface area of alveoli was significantly reduced in bleomycin injured lungs ventilated with PEEP = 1 cmH_2_O (Figure 4A and Table 1) indicates that the mechanical properties of the interalveolar septa were altered due to the mechanical ventilation of lungs suffering from microatelectases, independent of the surface tension at the air-liquid interface. At the electron microscopic level we observed interstitial edema at the thick side of the blood-gas barrier where the epithelial and endothelial basal laminae are separated by interstitial tissue including collagen fibrils and fibroblasts (Figure 6). Here, a diverging of collagen fibrils was seen. At light microscopic level this observation was linked with an increase of the mean septal wall thickness. Hence, an interstitial edema-related stiffening of the inter-alveolar septa is assumed to occur due to a disarrangement of collagen fibrils.

The presented data suggest a causal relationship between the existence of microatelectases during mechanical ventilation and the progression of VILI, although they do not provide a definite proof. This causality is supported by theoretical considerations using spring models to simulate the distribution of septal stresses and strains within a network of elastic elements (Mead et al., 1970; Makiyama et al., 2014; Albert et al., 2019). As long as the mechanical properties of the spring elements are similar the distribution of stresses and strains, and therefore the tissue deformation, is homogenous. In presence of springs with increased stiffness e.g., due to alveolar derecruitment the stresses on surrounding elements have been calculated to be increased 16-fold (Makiyama et al., 2014). Albert and co-workers simulated the strain of alveoli adjacent to derecruited alveoli and found that in dependence of the number of derecruited alveoli (which was in that model between 1 and 7) there could be an increase in septal wall strain of 30 to 50% at resting volume (Albert et al., 2019). Those predictions were in agreement with previous physiology-based, non-linear multi-compartment model simulations of mean static and dynamic alveolar strain during mechanical ventilation in bleomycin-treated rats at PEEPs where microatelectases were observed in tissue sections (Knudsen et al., 2018). Taken together, those simulations suggest that although there is no increased strain at the organ scale there might be local increased dynamic and static strain of a cohort of alveoli due to stress concentration in a lung which suffers from microatelectases (=group of derecruited alveoli).

There are several imaging studies which support the concept of the injurious potential of microatelectases creating locally increased strain via alveolar interdependence. Using functional magnetic resonance imaging (MRI) to determine the apparent diffusion coefficient (ADC), evidence for airspace overdistension in close proximity to atelectasis due to low PEEP ventilation or surfactant removal was established (Cereda et al., 2013, 2016). The ADC correlates very well with the mean linear intercept length of acinar airspaces measured at light microscopic level (Woods et al., 2006). However, the mean linear intercept length has to be interpreted with caution in terms of alveolar pathophysiology since it depends on both the dimension of the ductal and the alveolar airspaces (Knudsen et al., 2010). The use of the mean linear intercept length or ADC as a marker for alveolar or septal wall strain is therefore not appropriate and it is difficult to conclude whether or not there is injurious septal wall strain due to atelectases from those studies. Nevertheless, in spite of normal strain at the organ level, increased regional tidal strain of distal airspaces, as measured from the distribution of a tracer by means of positron emission tomography, has been shown to correlate with the degree of neutrophilic inflammation after invasive mechanical ventilation indicating induction of regional ventilation-induced lung injury (Wellman et al., 2014). Using an *ex vivo* approach and confocal microscopy to resolve individual alveolar septa, Wu and co-workers directly analyzed the effects of alveolar interdependence during mechanical ventilation by observing the interactions between an alveolus filled with protein-rich edema and a healthy alveolus (Wu et al., 2014). Although the strain at the organ scale was considered to be harmless, injury of the healthy alveolus, as measured by leakage of tracer into alveolar airspace, was induced and correlated to surface tension in the flooded alveolus (Wu et al., 2014).

Dolinay and co-workers demonstrated in a primary alveolar epithelial cell culture model that a bi-axial strain of 25%, which corresponds to ventilation with 15 ml/kg bodyweight (Tschumperlin and Margulies, 1999), the unfolded protein response is activated in a Ca^2+^ dependent manner. These findings could be reproduced *in vivo* in a rat model ventilated with a tidal volume of 20 ml/kg bodyweight and an *ex vivo* porcine model ventilated with a tidal volume of 15 ml/kg bodyweight (Dolinay et al., 2017, 2018). The investigators suggested that a stretch-induced cytosolic increase in Ca^2+^ concentration resulted in an autophosphorylation of Perk which in a further step phosphorylated EIF-2α. By p-Perk inhibition experiments, the authors also demonstrated that this pathway was of relevance for dysfunction of the blood-gas barrier resulting in a leakage of proteins into the alveolar space. In the present study, we used immunohistochemistry to localize phosphorylated EIF-2α within lung parenchyma and observed that in the healthy lung there was hardly any staining while after bleomycin challenge, approximately 23% of the alveolar surface area was covered by positive cells. While during PEEP 5 cmH_2_O this fraction remained roughly stable, PEEP = 1 cmH_2_O ventilation increased that fraction up to 35%. Of note, positive cells were predominantly observed in adjacent to microatelectases so that increased static and dynamic strain of alveoli appears to be a plausible explanation for this observation. Furthermore, this observation suggests that microatelectases could be the spatiotemporal origin for spreading of the integrated stress response.

The present study has several limitations. Based on the functional data described in this study the bleomycin injured lungs did not develop a severe respiratory failure after 3 h of mechanical ventilation with PEEP = 1 cmH_2_O. The transcutaneously measured oxygen saturation did not illustrate a substantial desaturation although lung mechanical properties as well as structural and BAL parameters measurably deteriorated. Hence, long term experiments expanding the duration of mechanical ventilation to allow further worsening of lung function are necessary to embed the relevance of our observations into the context of ARDS. As an alternative the PEEP could be reduced to zero (=ZEEP). This adjustment in the experimental protocol would result in an aggravation of microatelectases (Knudsen et al., 2018) and could potentially increase the vulnerability to ventilation-induced lung injury in this animal model even with shorter periods of mechanical ventilation. Moreover, in order to monitor the mechanical ventilation we used the oxygen saturation which demonstrated for all study groups under the given conditions sufficient gas exchange. However, we did not monitor arterial partial pressure of CO_2_, bicarbonate and pH during mechanical ventilation which would have provided an additional assessment of ventilation adequacy. While the measured oxygen saturation at room air makes a relevant hypoventilation unlikely we cannot exclude a hyperventilation with subsequent respiratory alkalosis which as such might have had an impact on our read-out parameters. However, the volumes of ventilated air per minute were comparable between the different PEEP levels and study groups (900 ml/min/kg bodyweight). Based on ultrastructural criteria, we quantified the degree of injury of the alveolar epithelial cells in lungs fixed by airway instillation and observed that after bleomycin challenge without mechanical ventilation approximately 42% of the epithelial basal lamina was covered by cells which showed ultrastructural criteria for injury. Using exactly the same stereological approach for quantification of alveolar epithelial injury (Lutz et al., 2015) used lung tissue fixed via vascular perfusion and found one day after bleomycin instillation a surface fraction of injured alveolar epithelium of 27%. Hence, the route of fixation might have had an impact on this parameter in a way that airway instillation of the fixative e.g., via fluid-induced shear stress aggravated the ultrastructural signs of epithelial injury in the present study.

In conclusion, we could demonstrate that bleomycin-induced microatelectases, which are hard to detect using organ-scale lung mechanical and functional data, are linked with an increased susceptibility for VILI during ventilation with tidal volumes that are not injurious to the initially healthy lung. Through the mechanism of alveolar interdependence these microatelectases might elevate local septal strains (in absence of abnormal strain at the organ-scale) to cause volutrauma of adjacent open alveoli which is characterized by progressive injury of the blood-gas barrier, reduced recruitability, and increased instability of distal airspaces. The role of the activated integrated stress response in the tethering-induced expansion of microatelectases and the progression of VILI warrants further investigation.

## Data Availability Statement

All datasets generated for this study are included in the article/Supplementary Material. The original virtual microscopic slides which were investigated in this study are also available as a repository under the first reference in the reference list: Albert et al. (2020).

## Ethics Statement

The animal study was reviewed and approved by Niedersächsisches Landesamt für Verbraucherschutz und Lebensmittelsicherheit (LAVES, Oldenburg, Lower Saxony, Germany, approval number 17/2068).

## Author Contributions

KA contributed to the conception and design, acquisition of data, analysis and interpretation of data, drafting and revising of the manuscript. J-MK contributed to the conception and design, acquisition of data, analyses and interpretation of data, revising of the manuscript. AP contributed to the conception and design, analysis and interpretation of data, revising of the manuscript. SW contributed to the conception and design, acquisition, analysis and interpretation of data, revising of the manuscript. EL-R contributed to the conception and design, analysis and interpretation of data, revising of the manuscript. AB contributed to the conception and design, analysis and interpretation of data, revising of the manuscript. BS contributed to the conception and design, analysis and interpretation of data, drafting and revising of the manuscript. LK contributed to the conception and design, acquisition of data, analysis and interpretation of data, drafting and revising of the manuscript.

## Conflict of Interest

The authors declare that the research was conducted in the absence of any commercial or financial relationships that could be construed as a potential conflict of interest.
