# Supplementary material for: Hidden Microatelectases Increase Vulnerability to Ventilation-Induced Lung Injury
Source: Front Physiol. 2020 Sep 18;11:530485. doi: 10.3389/fphys.2020.530485 (PMC7530907; doi:10.3389/fphys.2020.530485)
Supplement: Supplementary file 1 [file Table_1.DOCX]

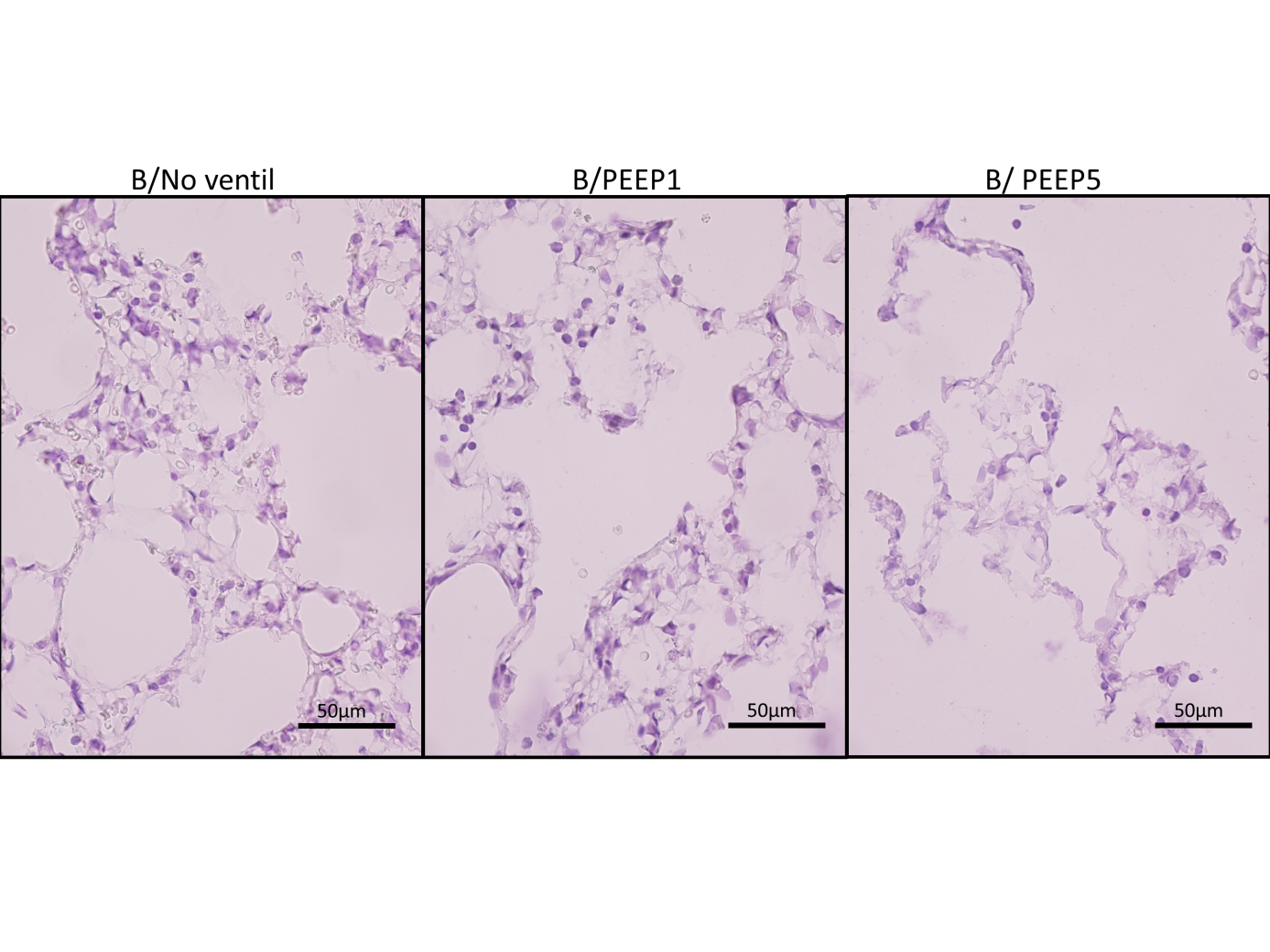


Suppl. Figure 1: Staining using isotype control antibody (Rabbit mAb IgG, Cell Signaling, #3900) as primary antibody at the same final concentration (2.205 µg/ml) as the Rabbit anti p-Perk mAb (Cell Signaling, #3179). After bleomycin challenge with and without mechanical ventilation there is no sign of unspecific binding of the primary antibody. This isotype control staining was repeated using the identical concentration as used for the Rabbit anti p-EIF-2α mAb (0.185 µg/ml, Cell Signaling # 3398) confirming the results illustrated in this figure.
